# Maternal Oxidative Balance Score during Pregnancy and Congenital Heart Defects

**DOI:** 10.3390/nu16121825

**Published:** 2024-06-11

**Authors:** Jiaomei Yang, Qiancheng Du, Ziqi Xiao, Rui Guo, Qianqian Chang, Yue-Hua Li

**Affiliations:** 1 Fourth Department of General Surgery, The Second Affiliated Hospital of Xi’an Jiaotong University, Xi’an 710004, China; 2 Department of Epidemiology and Biostatistics, School of Public Health, Xi’an Jiaotong University Health Science Center, Xi’an 710061, China; 3 Key Laboratory of Environment and Genes Related to Diseases, Ministry of Education, Xi’an Jiaotong University, Xi’an 710061, China

**Keywords:** oxidative balance score, congenital heart defects, pregnancy

## Abstract

The relationship between maternal oxidative balance score (OBS) in pregnancy, representing overall oxidative balance status by integrating dietary and lifestyle factors, and congenital heart defects (CHD) remains unclear; therefore, this study attempted to explore their associations among the Chinese population. We conducted a case-control study including 474 cases and 948 controls in Northwest China. Pregnant women were interviewed to report diets and lifestyles in pregnancy by structured questionnaires. Logistic regression models were used to estimate the adjusted ORs (95%CIs). Maternal OBS ranged from 6 to 34 among cases, and 5 to 37 among controls. Comparing the highest with the lowest tertile group, the adjusted OR for CHD was 0.31 (0.19–0.50). The CHD risk was reduced by 7% (OR = 0.93, 95%CI = 0.90–0.95) in association with per 1 higher score of OBS during pregnancy. The inverse relationship between maternal OBS and CHD risk appeared to be more pronounced among participants in urban areas (OR = 0.89, 95%CI = 0.86–0.93). Maternal OBS during pregnancy showed good predictive values for fetal CHD, with the areas under the receiver operating characteristic curve 0.78 (0.76–0.81). These findings highlighted the importance of reducing oxidative stress through antioxidant-rich diets and healthy lifestyles among pregnant women to prevent fetal CHD.

## 1. Introduction

Congenital heart defects (CHD) are the most common birth defects, affecting nearly 1 in 100 newborns both in the world [1] and in China [2]. CHD are among the leading causes of morbidity and mortality from congenital disorders, and cause over 0.2 million deaths worldwide each year [3], imposing substantial burdens on the family and society. However, the mechanisms of CHD remains largely unclear [4]. Therefore, it is important to identify modifiable risk factors for CHD to implement effective primary prevention to reduce the CHD incidence.

Previous studies have shown some modifiable exposures during pregnancy in association with CHD risk, such as maternal smoking [5], alcohol drinking [6], BMI [7], physical activity [8], and dietary habits [9,10,11,12,13,14]. These exogenous exposures have either oxidative or antioxidative effects in maternal body, which may further influence fetal cardiovascular development through reactive oxygen species and redox-related signaling [15]. For example, some nutrients (e.g., vitamin C, vitamin E, selenium, and zinc) and lifestyles (e.g., physical activity) are antioxidants, while some nutrients (e.g., total fat and iron) and lifestyles (e.g., smoking, alcohol drinking, and BMI) are prooxidants [16]. However, previous studies on these exposures and CHD typically focus on single exposure, without considering the potential cumulative effects of oxidation and antioxidation. The oxidative balance score (OBS) has been developed that integrates multiple dietary and lifestyle factors known to influence oxidative stress and represents their potential cumulative effects [16], with higher OBS indicating lower oxidative stress [16]. This simple scoring system is important because it is easy to be understood and translated into practice with great public health implications. Emerging studies have reported that higher OBS was associated with reduced risks of chronic diseases such as type 2 diabetes [17], cardiovascular diseases [18], cancers [19], hypertension [20], and obesity [21]. However, there are few studies exploring maternal OBS in relation with pregnancy outcomes [15,22,23]. To the best of our knowledge, there has been no study specially investigating the relationship between maternal OBS in pregnancy and CHD. Prior researches have reported some indices during pregnancy for the early prediction of CHD [9,10]. However, no studies have evaluated the potential predictive value of maternal OBS during pregnancy on CHD.

In this case-control study in Northwest China, we attempted to examine the association between maternal OBS during pregnancy and CHD risk, and evaluate the predictive value for OBS on CHD.

## 2. Materials and Methods

### 2.1. Study Design and Population

From August 2014 to August 2016, we conducted a case-control study in six cooperative hospitals in Xi’an City, Northwest China. The study design has been reported in detail previously [11,13,14]. Briefly, we recruited pregnant women awaiting delivery in hospitals. We included participants having fetuses with isolated CHD and no genetic disorders in the case group, and those having normal fetuses without any birth defects in the control group. We excluded participants with multiple pregnancies or diabetes due to the potential distinct etiologies. No mothers were diagnosed with cardiovascular or autoimmune diseases in the included participants. Qualified professionals from ultrasound, obstetrics, and pediatrics departments in each hospital strictly enforced the standard diagnostic criteria. All cooperative hospitals have integrated CHD screening into routine prenatal and postnatal check. Fetuses received echocardiography at 20th–24th gestational week, and newborns received cardiac auscultation and percutaneous pulse oxygen saturation measurement within 72 h after birth. To differentiate atrial septal defects (ASD) and patent foramen ovale, ultrasound professionals carefully identified whether the continuity of atrial septal was intact and the foramen ovale was open in various sections of fetal atrial septal through echocardiography. We further undertook a telephone follow-up within one year after birth to confirm the above diagnoses. Those fetuses who had patent foramen ovale but closed the foramen ovale within one year after birth were not categorized as CHD. We randomly selected controls in each hospital each month to meet the ratio of the number of controls to cases in the same hospital in the same month 2:1. To detect a significant (*p* < 0.05) OR of 0.75 between the high and low OBS groups with a statistical power of 80%, 443 cases and 886 controls would be required. A total of 474 cases and 948 controls were included in the final analyses, meeting the sample size requirements.

This study was approved by the Xi’an Jiaotong University Health Science Center (No. 2012008, date: 3 March 2012). All women provided informed consent prior to the study.

### 2.2. Dietary Assessment and Oxidative Balance Score

We collected maternal diets throughout pregnancy by a 111-item semi-quantitative food frequency questionnaire (FFQ) developed for pregnant women in Northwest China [24]. Maternal dietary habits tend to be stable across pregnancy [25]; thus, their diets throughout pregnancy are comparable with those in the 3rd–8th gestational week, the critical period of fetal heart development [11,12,13,14]. We used the Food Composition Tables in China to derive nutrient intakes from diets [26,27]. Women also recalled the information on dietary supplements in each trimester of pregnancy, including the type/brand and the number of supplements and days they took. The intake of each nutrient was calculated as the sum from diets and dietary supplements.

We included 16 nutrients and 4 lifestyle factors in the OBS components according to previous studies [28,29,30]. These components were classified as 15 antioxidants (fiber, β-carotene, vitamin B_2_, niacin, vitamin B_6_, folate, vitamin B_12_, vitamin C, vitamin E, calcium, magnesium, zinc, copper, selenium, and physical activity) and 5 prooxidants (total fat, iron, smoking, alcohol drinking, and BMI). Information on physical activity, active/passive smoking, and alcohol drinking in early pregnancy was obtained by a structured questionnaire. Prepregnancy weight and height were reported by pregnancy women to calculate BMI. The details of the assignment scheme for OBS are shown in Table S1. Nutrient intakes and BMI were divided into 3 groups according to tertiles of the control distribution. From the first to the third tertile, the antioxidants were assigned scores from 0 to 2, and the prooxidants were assigned scores from 2 to 0. For physical activity, participants reporting inactive, low, and moderate/high activity received 0, 1, and 2 points, respectively. For smoking, participants reporting active smoking/passive smoking without avoidance measures, passive smoking with avoidance measures, and none received 0, 1, and 2 points, respectively. For alcohol drinking, participants reporting ≥3 times/week, <3 times/week, and none received 0, 1, and 2 points, respectively. The OBS was calculated by summing the scores for these 20 components, with a higher OBS indicating greater exposure to antioxidants.

### 2.3. Covariates

Trained investigators collected general information of participants by a structured questionnaire. The study covariates included maternal age (<30 years/≥30 years), education (junior high school or below/senior high school or above), occupation (in employment/without employment), residence (rural/urban), parity (0/≥1), and medication use (no/yes) and anemia (no/yes) in early pregnancy. Women with no paid employment outside their homes were classified as without employment. Women with hemoglobin concentration < 110 g/L were diagnosed with anemia in pregnancy.

### 2.4. Statistical Analyses

In univariate comparisons, we compared categorical variables by χ^2^ test or Fisher’s exact test, and continuous variables by Mann–Whitney U test or Kruskal–Wallis test due to the non-normal distributions. Considering the clustering in the design through hospitals, we applied mixed logistic regression models to estimate ORs (95%CIs) for total CHD and CHD subtypes associated with maternal OBS in pregnancy. We divided maternal OBS into three groups according to tertiles of the control distribution. The confounders were adjusted in the models according to previous studies [11,13,31] and the change in estimates by over 10% [32], which finally included maternal age, education, occupation, residence, parity, medication use, anemia, and total energy intake. *p* for trend was calculated by including the medians to each tertile in the model. We stratified the analyses by maternal characteristics (age, education, occupation, residence, parity, medication use, and anemia), and assessed effect modification between each subgroup factor and OBS by testing an interaction product-term with the likelihood ratio test. We further conducted sensitivity analyses by removing one component from the total OBS score at a time and including this component in the model as a covariate.

We constructed the receiver operating characteristic curves (ROC) to estimate the optimal cut-off value of OBS in pregnancy for CHD with the maximum Youden index. The areas under the ROC (AUC) showed the accuracy of OBS as a predictor for CHD, with the AUC values indicating the predictive power as follows: >0.7, useful; >0.8, good; and >0.9, very good [33].

All analyses were undertaken with Stata version 15.0 (StataCorp, College Station, TX, USA). Two-sided *p* < 0.05 was considered as statistically significant.

## 3. Results

### 3.1. Baseline Characteristics of the Study Participants

**Figure 1:**
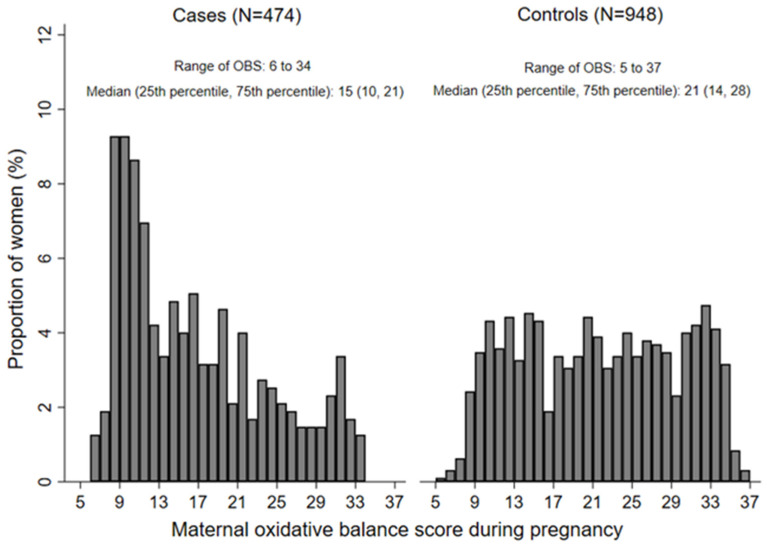
The distribution of maternal oxidative balance score during pregnancy among cases and controls. OBS, oxidative balance score.

Among the 474 CHD babies, 222 had VSD, 218 had ASD, and less than 100 had other specific CHD subtypes such as patent ductus arteriosus, atrioventricular septal defects, and tetralogy of fallot. Figure 1 shows the distribution of maternal OBS during pregnancy. Maternal OBS ranged from 6 to 34 among cases, and 5 to 37 among controls. The medians (25th percentile, 75th percentile) among cases and controls were 15 (10, 21) and 21 (14, 28), respectively, with the difference statistically significant (*p* < 0.001). Table 1 displays the baseline characteristics by tertiles of OBS. Pregnant women with higher OBS tended to have higher education levels in both cases and controls. Participants with higher OBS were more likely to reside in rural areas among cases and reside in urban areas among controls. Case mothers with higher OBS also tended to be nullparity. There were significant differences in maternal education, occupation, residence, parity, and medication use and anemia in early pregnancy among cases and controls (all *p* < 0.05) (Table S2).

### 3.2. The Distribution of OBS Components among Groups

Pregnant women with higher OBS had higher intakes of all dietary OBS components but lower proportion of active/passive smoking both among cases and controls (all *p* < 0.001) (Table 2). Participants with higher OBS showed lower BMI among cases (*p* = 0.024). Compared with those in controls, pregnant women in cases had lower intakes of all dietary OBS components but higher proportion of alcohol drinking and higher BMI (all *p* < 0.001) (Table S3).

### 3.3. Association between Maternal OBS during Pregnancy and CHD

The risks for total CHD, ventricular septal defects (VSD), and ASD were reduced with increasing tertile of maternal OBS in pregnancy, and the tests for trend were statistically significant (all *p* for trend < 0.001) (Table 3). Comparing participants in the highest (third) tertile group with those in the lowest (first) tertile group, the adjusted ORs (95%CIs) for total CHD, VSD, and ASD were 0.31 (0.19–0.50), 0.37 (0.23–0.57), and 0.42 (0.27–0.64), respectively. The risks for total CHD, VSD, and ASD were reduced by 7% (OR = 0.93, 95%CI = 0.90–0.95), 7% (OR = 0.93, 95%CI = 0.89–0.96), and 8% (OR = 0.92, 95%CI = 0.89–0.95) in association with per 1 higher score of maternal OBS during pregnancy, respectively.

Subgroup analyses showed that the associations of OBS in pregnancy with total CHD, VSD, and ASD did not materially change by maternal age, education, occupation, parity, or medication use and anemia in early pregnancy (Figures S1–S3). However, the inverse relationship appeared to be more pronounced among participants in urban areas, with all *P* for interaction < 0.003 (Figures S1–S3). After removing each OBS component at a time and adjusting for the removed one as a covariate, the inverse relationship between OBS in pregnancy and CHD remained significant and barely changed (a maximum difference of 3.9% comparing with the primary analysis) (Figures S4–S6).

### 3.4. The Prediction Value for Maternal OBS in Pregnancy on CHD

**Figure 2:**
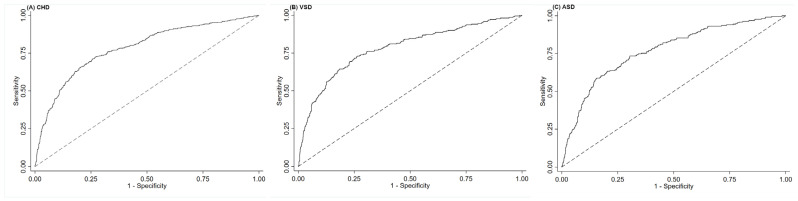
The ROC for maternal oxidative balance score in pregnancy in the prediction of (**A**) total congenital heart defects, (**B**) ventricular septal defects, and (**C**) atrial septal defects. ASD, atrial septal defects; CHD, congenital heart defects; ROC, receiver operating characteristic curves; VSD, ventricular septal defects. The dotted line refers to the reference line that results from random selection.

The ROC suggested that maternal OBS was useful to predict total CHD, VSD, and ASD, with the AUC to be 0.78 (0.76, 0.81), 0.78 (0.75, 0.82), and 0.77 (0.73, 0.81), respectively (Figure 2). The optimal OBS cut-off values were 16 for total CHD (specificity: 74.3%, sensitivity: 71.3%), 13 for VSD (specificity: 73.7%, sensitivity: 73.0%), and 15 for ASD (specificity: 79.8%, sensitivity: 63.3%), respectively.

## 4. Discussion

In the present study, we observed that maternal higher OBS during pregnancy, indicating lower oxidative stress due to dietary and lifestyle factors, was related with lower risks of total CHD, VSD, and ASD. These inverse relationships seemed to be more pronounced among pregnant women in urban areas. We also found that maternal OBS in pregnancy had good predictive values for fetal CHD, VSD, and ASD. To the best of our knowledge, this is the first human study to specially investigate the effects of maternal OBS in pregnancy on fetal CHD.

To date, few human studies have assessed the associations of maternal OBS in pregnancy with birth outcomes. There are two prior studies reporting maternal OBS in pregnancy as a contributor to limb deficiencies and other neural crest cell-related congenital anomalies using data from the National Birth Defects Prevention Study in America [15,22]. These two studies constructed the OBS using different components, one emphasizing on the dietary intake [22] and another combining both dietary intake and lifestyle factors [15], but reported consistent results on the associations of individual OBS components with limb deficiencies [15,22]. Our study constructed the OBS by choosing dietary and lifestyle components that have known influence on oxidative balance as well as CHD [5,6,7,8,12,16], which was widely used among emerging studies on nonperinatal outcomes such as chronic diseases and sleep quality [17,18,19,28]. Our results was supported by previous studies on the associations of individual OBS components with CHD, such as smoking [5], alcohol drinking [6], BMI [7], physical activity [8], and antioxidant nutrients [12]. In addition, we have previously used other scoring approaches including the Global Diet Quality Score, Mediterranean Diet Score, and Dietary Inflammation Index to assess the overall dietary quality and dietary inflammatory potential with CHD risk rather than just examining one nutrient at a time [9,10]. The OBS approach is similar, and would extend the previous reports that only examine one exposure at a time and reflect how multiple exposures jointly influence outcomes. It has been reported that maternal OBS in pregnancy could serve as a valid reflective indicator of urinary F2-isoprostane, an objective indicator of oxidative stress [23]. Therefore, the OBS could provide an easy and noninvasive way to evaluate maternal oxidative stress status in pregnancy as a predictor for fetal CHD. The current study implies that it is warranted to integrate the suggestion of reducing oxidative stress through multiple antioxidant-rich diets and healthy lifestyles in the routine pregnancy management practices to prevent the occurrence of CHD.

Pregnant women usually have increased susceptibility to oxidative stress, with the elevated presence of reactive oxygen species and reactive nitrogen species in the body [34]. The state of excessive oxidation can cause oxidative damage involving nucleic acids, proteins, lipids, and carbohydrates with complex mechanisms [35]. Previous studies have demonstrated that reactive oxygen species is important in embryonic development because of the influence in cell signaling pathways involving proliferation, differentiation, and apoptosis [36]. Some prooxidants in pregnancy can increase the oxidative stress, while antioxidants can obviate these effects through modification of gene expression, cell cycle alterations, and transcription factor signaling [36]. Animal studies found that some prooxidants in pregnancy caused higher oxidative stress levels and changed key cardiogenic regulator expression in the developing heart [37]. One prior study indicated that pregnant women with fetal CHD had higher levels of oxidative stress biomarkers than those with normal fetuses, emphasizing the importance of the balance between oxidative stress and antioxidant defense in fetal heart development [38]. In addition, oxidative stress in pregnancy may be accompanied by systemic inflammatory response [39], which may disrupt embryonic heart development by the increased pro-inflammatory cytokines [40].

The present study provides important evidence on CHD risk associated with maternal OBS in pregnancy. However, some limitations should be discussed. Firstly, maternal information in pregnancy was reported by pregnant women awaiting delivery, which may bring recall bias. However, prior studies have indicated that mothers could recall information during pregnancy well after years [41,42]. Secondly, dietary data in the whole pregnancy rather than the critical period of fetal heart development in early pregnancy was gathered, which may bring exposure misclassification. However, prior studies have indicated that dietary habits in pregnancy were generally stable throughout pregnancy [25]. Thirdly, CHD fetuses that did not survive before delivery were not included in this study and both cases and controls were recruited from hospitals, which may bring selection bias. Fourthly, the impacts of maternal OBS with other CHD subtypes were not assessed, given the sample size limitation. Finally, residual confounders from unobserved or unknown factors may exist, and the real causal relationship cannot be derived from the observational study.

## 5. Conclusions

This case-control study suggested that maternal higher OBS in pregnancy, indicating lower oxidative stress due to dietary and lifestyle factors, was associated with lower risk of CHD. This study also suggested maternal OBS during pregnancy had good predictive values for fetal CHD. These findings highlighted the importance of reducing oxidative stress through antioxidant-rich diets and healthy lifestyles among pregnant women to prevent the incidence of CHD.

## Figures and Tables

**Table 1:** Baseline characteristics of the study participants according to tertiles of maternal oxidative balance score in pregnancy ^1^.

	Cases (*N* = 474)				Controls (*N* = 948)			
	Tertile 1 (*N* = 279)	Tertile 2 (*N* = 124)	Tertile 3 (*N* = 71)	*p* ^2^	Tertile 1 (*N* = 316)	Tertile 2 (*N* = 303)	Tertile 3 (*N* = 329)	*p* ^2^
Oxidative balance score								
Range	6.0 to 16.0	17.0 to 25.0	26.0 to 34.0		5.0 to 16.0	17.0 to 25.0	26.0 to 37.0	
Median (25th percentile, 75th percentile)	10.0 (9.0, 13.0)	20.5 (19.0, 23.0)	30.0 (28.0, 31.0)	<0.001	12.0 (10.0, 14.0)	21.0 (19.0, 23.0)	31.0 (28.0, 32.0)	<0.001
Baseline characteristics, *N* (%)								
Maternal age ≥ 30 years	98 (35.1)	37 (29.8)	24 (33.8)	0.583	110 (34.8)	98 (32.3)	116 (35.3)	0.712
Maternal education, senior high school or above	147 (52.7)	80 (64.5)	52 (73.2)	0.002	235 (74.4)	240 (79.2)	290 (88.1)	<0.001
Maternal occupation, in employment	131 (47.0)	65 (52.4)	44 (62.0)	0.070	235 (74.4)	246 (81.2)	266 (80.9)	0.061
Rural residence	78 (28.0)	45 (36.3)	38 (53.5)	<0.001	108 (34.2)	89 (29.4)	72 (21.9)	0.002
Nullparity	149 (53.4)	71 (57.3)	54 (76.1)	0.003	247 (78.2)	244 (80.5)	270 (82.1)	0.457
Medication use in early pregnancy	117 (41.9)	52 (41.9)	28 (39.4)	0.925	83 (26.3)	93 (30.7)	112 (34.0)	0.099
Anemia in early pregnancy	52 (18.6)	22 (17.7)	6 (8.5)	0.118	40 (12.7)	31 (10.2)	32 (9.7)	0.446

^1^ The three groups for oxidative balance score was classified by tertiles of the control distribution. ^2^ Categorical variables were compared by χ^2^ test and continuous variables by Kruskal-Wallis test.

**Table 2:** Oxidative balance score components according to tertiles of maternal oxidative balance score in pregnancy.

	Cases (*N* = 474)				Controls (*N* = 948)			
	Tertile 1	Tertile 2	Tertile 3	*p* ^1^	Tertile 1	Tertile 2	Tertile 3	*p* ^1^
OBS components ^2^								
Dietary OBS components ^3^								
Fiber, g/d	15.3 (12.7, 18.6)	23.6 (20.5, 28.8)	37.7 (30.0, 46.5)	<0.001	13.4 (11.0, 16.4)	21.4 (17.1, 25.0)	33.5 (25.2, 45.1)	<0.001
β-Carotene, RE/d	1065.4 (766.2, 1503.2)	2292.9 (1576.3, 3620.6)	4606.3 (2778.1, 6582.8)	<0.001	1075.5 (783.2, 1299.0)	1866.8 (1288.2, 2349.0)	3158.9 (2190.2, 6152.9)	<0.001
Vitamin B_2_, mg/d	0.4 (0.3, 0.6)	0.8 (0.7, 1.0)	1.4 (1.1, 2.0)	<0.001	0.5 (0.4, 0.7)	0.9 (0.8, 1.1)	1.7 (1.2, 2.3)	<0.001
Niacin, mg/d	8.1 (6.1, 9.7)	12.6 (10.3, 14.9)	21.0 (16.5, 29.6)	<0.001	8.4 (6.6, 10.4)	13.5 (11.6, 16.3)	22.9 (18.3, 30.4)	<0.001
Vitamin B6, mg/d	0.4 (0.3, 0.5)	0.8 (0.6, 0.9)	1.3 (1.0, 1.8)	<0.001	0.4 (0.3, 0.5)	0.7 (0.6, 0.8)	1.4 (1.0, 2.1)	<0.001
Folate, mg/d	150.3 (96.9, 198.6)	259.2 (199.4, 341.6)	406.0 (302.8, 541.7)	<0.001	205.4 (160.7, 263.4)	272.1 (232.9, 372.4)	446.4 (344.3, 621.7)	<0.001
Vitamin B_12_, mg/d	0.1 (0, 0.2)	0.1 (0, 0.4)	0.3 (0.1, 1.4)	<0.001	0.2 (0.1, 0.3)	0.3 (0.1, 0.7)	1.0 (0.3, 2.4)	<0.001
Vitamin C, mg/d	47.0 (33.0, 64.7)	98.4 (73.1, 122.7)	173.6 (136.9, 229.1)	<0.001	50.2 (39.9, 64.3)	86.6 (69.1, 111.1)	184.2 (125.0, 302.8)	<0.001
Vitamin E, mg/d	8.3 (5.3, 11.8)	16.6 (12.0, 21.2)	26.9 (21.9, 34.3)	<0.001	9.2 (6.9, 12.8)	16.2 (13.3, 20.6)	30.2 (22.5, 40.7)	<0.001
Calcium, mg/d	328.6 (210.0, 445.1)	566.0 (458.0, 694.7)	1010.3 (762.9, 1213.4)	<0.001	437.3 (324.2, 553.8)	618.5 (506.8, 748.1)	946.8 (755.3, 1191.8)	<0.001
Magnesium, mg/d	155.3 (122.2, 206.8)	278.6 (240.9, 336.7)	452.2 (373.6, 556.3)	<0.001	158.1 (132.0, 198.1)	262.5 (221.9, 304.9)	435.5 (344.4, 584.6)	<0.001
Zinc, mg/d	3.6 (2.5, 4.6)	6.72 (5.85, 7.74)	10.8 (9.5, 15.0)	<0.001	4.4 (3.5, 5.2)	7.2 (6.0, 8.5)	12.9 (10.0, 16.8)	<0.001
Copper, mg/d	1.4 (0.9, 1.7)	2.0 (1.5, 2.3)	2.8 (2.2, 3.6)	<0.001	1.2 (0.9, 1.7)	2.0 (1.6, 2.3)	3.0 (2.5, 3.8)	<0.001
Selenium, mg/d	16.9 (12.2, 23.3)	30.1 (24.0, 37.6)	48.2 (37.1, 62.2)	<0.001	20.8 (16.7, 24.9)	32.6 (26.0, 38.3)	51.7 (40.3, 72.2)	<0.001
Total fat, g/d	21.3 (15.0, 34.0)	40.9 (28.8, 54.4)	60.9 (46.9, 77.8)	<0.001	27.6 (20.8, 36.3)	43.7 (32.0, 54.5)	62.0 (47.0, 83.3)	<0.001
Iron, mg/d	15.2 (10.2, 19.4)	22.2 (18.0, 28.5)	34.7 (26.9, 49.2)	<0.001	17.6 (12.3, 22.7)	25.3 (20.8, 33.9)	41.8 (30.8, 55.1)	<0.001
Lifestyle OBS components								
Moderate/high physical activity	15 (5.4)	14 (11.3)	4 (5.6)	0.078	25 (7.9)	22 (7.3)	33 (10.0)	0.420
No smoking	146 (52.3)	81 (65.3)	52 (73.2)	<0.001	146 (46.2)	189 (62.4)	203 (61.7)	<0.001
No alcohol drinking	271 (97.1)	118 (95.2)	70 (98.6)	0.375	312 (98.7)	302 (99.7)	327 (99.4)	0.379
Body mass index, kg/m^2^	23.1 (20.2, 24.3)	23.2 (19.8, 24.0)	22.2 (19.1, 23.9)	0.310	21.3 (20.1, 23.8)	20.9 (19.6, 23.4)	20.7 (19.5, 22.7)	0.024

OBS, oxidative balance score. RE, retinol equivalent. ^1^ Categorical variables were compared by χ^2^ test/Fisher’s exact test and continuous variables by Kruskal-Wallis test. ^2^ Continuous variables are displayed as median (25th percentile, 75th percentile), and categorical variables as n (%). ^3^ The intake of each nutrient intake was calculated as the sum from diets and dietary supplements.

**Table 3:** Associations between maternal oxidative balance score in pregnancy and congenital heart defects.

	Tertile 1	Tertile 2	Tertile 3	*p*_trend_	Per 1 Higher Score
Total congenital heart defects					
*N*_cases_/*N*_controls_	279/316	124/303	71/329	474/948	474/948
Unadjusted OR (95%CI)	1	0.46 (0.36, 0.60)	0.24 (0.18, 0.33)	<0.001	0.92 (0.91, 0.94)
Adjusted OR (95%CI) ^1^	1	0.56 (0.41, 0.77)	0.31 (0.19, 0.50)	<0.001	0.93 (0.90, 0.95)
Ventricular septal defects					
*N*_cases_/*N*_controls_	131/316	58/303	33/329	222/948	222/948
Unadjusted OR (95%CI)	1	0.46 (0.33, 0.66)	0.24 (0.16, 0.37)	<0.001	0.92 (0.90, 0.94)
Adjusted OR (95%CI) ^1^	1	0.63 (0.43, 0.93)	0.37 (0.23, 0.57)	<0.001	0.93 (0.89, 0.96)
Atrial septal defects					
*N*_cases_/*N*_controls_	116/316	63/303	39/329	218/948	218/948
Unadjusted OR (95%CI)	1	0.57 (0.40, 0.80)	0.32 (0.22, 0.48)	<0.001	0.94 (0.92, 0.95)
Adjusted OR (95%CI) ^1^	1	0.70 (0.48, 1.02)	0.42 (0.27, 0.64)	<0.001	0.92 (0.89, 0.95)

^1^ Adjusted for total energy intake, maternal age, education, occupation, residence, parity, medication use, and anemia.

## Data Availability

The datasets in this study are available from the corresponding author on reasonable request.

## Supplementary Materials

The following supporting information can be downloaded at: https://www.mdpi.com/article/10.3390/nu16121825/s1, Table S1: Oxidative balance score assignment scheme; Table S2: Baseline characteristics of the study participants among cases and controls; Table S3: Oxidative balance score components among cases and controls; Figure S1: Subgroup analyses for the relationship between per 1 higher in maternal oxidative balance score in pregnancy and total congenital heart defects; Figure S2: Subgroup analyses for the relationship between per 1 higher in maternal oxidative balance score in pregnancy and ventricular heart defects; Figure S3: Subgroup analyses for the relationship between per 1 higher in maternal oxidative balance score in pregnancy and atrial heart defects; Figure S4: The relationship between per 1 higher in maternal oxidative balance score in pregnancy and total congenital heart defects after alternate subtraction of each component; Figure S5: The relationship between per 1 higher in maternal oxidative balance score in pregnancy and ventricular heart defects after alternate subtraction of each component; Figure S6: The relationship between per 1 higher in maternal oxidative balance score in pregnancy and atrial heart defects after alternate subtraction of each component.
