# Supplementary material for: Maternal Oxidative Balance Score during Pregnancy and Congenital Heart Defects
Source: Nutrients. 2024 Jun 11;16(12):1825. doi: 10.3390/nu16121825 (PMC11206902; doi:10.3390/nu16121825)
Supplement: Supplementary file 1 [file nutrients-16-01825-s001.zip › nutrients-2979922-supplementary.pdf]

Table S1. Oxidative balance score assignment scheme.

OBS components	Property	Score[1]		
Dietary OBS components[2]		0	1	2
Fiber, g/d	A	<16.30	16.30-25.44	≥25.44
β-Carotene, RE/d	A	<1201.87	1201.87-2360.24	≥2360.24
Vitamin B_2, mg/d	A	<0.71	0.71-1.17	≥1.17
Niacin, mg/d	A	<11.02	11.02-17.47	≥17.47
Vitamin B_6, mg/d	A	<0.52	0.52-0.93	≥0.93
Folate, mg/d	A	<237.44	237.44-378.19	≥378.19
Vitamin $B_{12,}$ mg/d	A	<0.15	0.15-0.52	≥0.52
Vitamin C, mg/d	A	<66.10	66.10-123.24	≥123.24
Vitamin E, mg/d	A	<12.95	12.95-22.43	≥22.43
Calcium, mg/d	A	<516.70	516.70-774.49	≥774.49
Magnesium, mg/d	A	<205.01	205.01-321.43	≥321.43
Zinc, mg/d	A	<5.74	5.74-9.38	≥9.38
Copper, mg/d	A	<1.60	1.60-2.42	≥2.42
Selenium, mg/d	A	<25.26	25.26-40.00	≥40.00
Total fat, g/d	P	≥52.45	32.98-52.45	<32.98
Iron, mg/d	P	≥34.15	21.16-34.15	<21.16
Lifestyle OBS components				
Physical activity	A	Inactive	Low	Moderate/high
Smoking	P	Active smoking/passive smoking without avoidance measures	Passive smoking with avoidance measures	None
Alcohol drinking	P	≥3 times/week	<3 times/week	None
Body mass index, kg/m^2	P	≥22.31	20.34-22.31	<20.34

A, antioxidant; OBS, oxidative balance score; P, prooxidant; RE, retinol equivalent.

[1] The three groups for continuous variables were categorized by tertiles of the control distribution.

[2] The intake of each nutrient intake was calculated as the sum from diets and dietary supplements.

Table S2. Baseline characteristics of the study participants among cases and controls.

	Cases (N = 474)	Controls (N = 948)	P[1]
Baseline characteristics, n (%)			
Maternal age ≥30 years	159 (33.5)	324 (34.2)	0.812
Maternal education, senior high school or above	279 (58.9)	765 (80.7)	<0.001
Maternal occupation, in employment	240 (50.6)	747 (78.8)	<0.001
Rural residence	161 (34.0)	269 (28.4)	0.030
Nullparity	274 (57.8)	761 (80.3)	<0.001
Medication use in early pregnancy	197 (41.6)	288 (30.4)	<0.001
Anemia in early pregnancy	80 (16.9)	103 (10.9)	0.001

[1] P values are from χ^2 test for categorical variables.

Table S3. Oxidative balance score components among cases and controls.

	Cases (N = 474)	Controls (N = 948)	P[1]
OBS components[2]			
Dietary OBS components[3]			
Fiber, g/d	19.2 (14.4, 25.7)	20.8 (14.2, 28.8)	0.022
β-Carotene, RE/d	1502.5 (951.0, 2754.3)	1799.6 (1083.2, 2690.4)	0.012
Vitamin B_2, mg/d	0.6 (0.4, 0.9)	0.9 (0.6, 1.4)	<0.001
Niacin, mg/d	9.9 (7.3, 13.7)	13.7 (9.7, 20.0)	<0.001
Vitamin B_6, mg/d	0.6 (0.4, 0.8)	0.7 (0.5, 1.2)	<0.001
Folate, mg/d	195.3 (127.5, 293.1)	293.8 (211.5, 426.6)	<0.001
Vitamin B_{12}, mg/d	0.1 (0.0, 0.2)	0.3 (0.1, 0.9)	<0.001
Vitamin C, mg/d	67.9 (42.4, 112.7)	88.2 (58.2, 160.7)	<0.001
Vitamin E, mg/d	11.8 (7.0, 18.6)	16.9 (10.9, 26.4)	<0.001
Calcium, mg/d	440.0 (293.3, 669.1)	636.2 (452.1, 879.1)	<0.001
Magnesium, mg/d	213.3 (145.2, 292.9)	258.0 (176.9, 382.4)	<0.001
Zinc, mg/d	5.1 (3.2, 7.3)	7.2 (5.1, 10.9)	<0.001
Copper, mg/d	1.6 (1.1, 2.1)	2.0 (1.4, 2.7)	<0.001
Selenium, mg/d	23.2 (15.3, 32.8)	32.5 (22.7, 46.9)	<0.001
Total fat, g/d	30.9 (19.1, 47.8)	41.7 (29.0, 59.5)	<0.001
Iron, mg/d	18.6 (13.3, 25.8)	27.0 (18.8, 40.6)	<0.001
Lifestyle OBS components			
Moderate/high physical activity	33 (7.0)	80 (8.4)	0.332
No smoking	279 (58.9)	538 (56.8)	0.448
No alcohol drinking	459 (96.8)	941 (99.3)	<0.001
Body mass index, kg/m^2	23.1 (20.0, 24.3)	21.0 (19.7, 23.3)	<0.001

RE, retinol equivalent.

[1] P values are from χ^2 test for categorical variables and from Mann–Whitney U test for continuous variables.

[2].Continuous variables are present as median (25th percentile, 75th percentile), and categorical variables are present as n (%).

[3] The intake of each nutrient intake was calculated as the sum from diets and dietary supplements.

Subgroups	$N_{(cases)}$	$N_{(controls)}$	OR (95%CI)	P for interaction
**Maternal age**				0.661
<30 years	315	624	0.94 (0.91, 0.97)	
≥ 30 years	159	324	0.91 (0.87, 0.95)	
**Maternal education**				0.521
Junior high school or below	195	183	0.94 (0.88, 1.01)	
Senior high shool or above	279	765	0.92 (0.89, 0.95)	
**Maternal occupation**				0.500
In employment	240	747	0.94 (0.91, 0.97)	
Without employment	234	201	0.92 (0.88, 0.96)	
**Residence**				<0.001
Urban	313	679	0.89 (0.86, 0.93)	
Rural	161	269	0.97 (0.92, 1.01)	
**Parity**				0.061
0	274	761	0.94 (0.91, 0.97)	
≥ 1	200	187	0.89 (0.85, 0.94)	
**Medication use**				0.101
No	277	660	0.94 (0.91, 0.98)	
Yes	197	288	0.90 (0.86, 0.94)	
**Anemia**				0.467
No	394	845	0.93 (0.90, 0.95)	
Yes	80	103	0.91 (0.85, 0.98)	
**Overall**	474	948	0.93 (0.90, 0.95)	

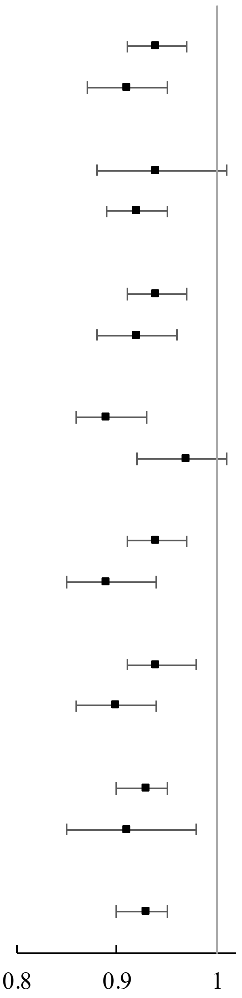

Figure S1. Subgroup analyses the relationship between per 1 higher in maternal oxidative balance score in pregnancy and total congenital heart defects. Analyses were adjusted for total energy intake, maternal age, education, occupation, residence, parity, medication use, and anemia.

Subgroups	$N_{(cases)}$	$N_{(controls)}$	OR (95%CI)	P for interaction
**Maternal age**				0.548
<30 years	150	624	0.96 (0.91, 1.00)	
≥ 30 years	72	324	0.87 (0.82, 0.93)	
**Maternal education**				0.783
Junior high school or below	97	183	0.94 (0.86, 1.03)	
Senior high shool or above	125	765	0.93 (0.89, 0.97)	
**Maternal occupation**				0.055
In employment	112	747	0.95 (0.91, 0.99)	
Without employment	110	201	0.89 (0.83, 0.94)	
**Residence**				0.002
Urban	158	679	0.91 (0.86, 0.96)	
Rural	64	269	0.96 (0.90, 1.02)	
**Parity**				0.067
0	124	761	0.96 (0.92, 1.01)	
≥ 1	98	187	0.87 (0.82, 0.94)	
**Medication use**				0.063
No	134	660	0.95 (0.91, 1.00)	
Yes	88	288	0.88 (0.83, 0.93)	
**Anemia**				0.682
No	190	845	0.93 (0.89, 0.97)	
Yes	32	103	0.88 (0.80, 0.97)	
**Overall**	222	948	0.93 (0.89, 0.96)	

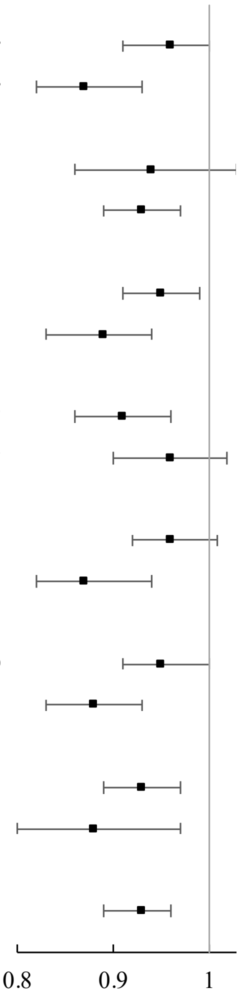

Figure S2. Subgroup analyses for the relationship between per 1 higher in maternal oxidative balance score in pregnancy and ventricular heart defects. Analyses were adjusted for total energy intake, maternal age, education, occupation, residence, parity, medication use, and anemia.

Subgroups	$N_{(cases)}$	$N_{(controls)}$	OR (95%CI)	P for interaction
**Maternal age**				0.548
<30 years	145	624	0.92 (0.88, 0.96)	
≥ 30 years	73	324	0.91 (0.86, 0.96)	
**Maternal education**				0.934
Junior high school or below	70	183	0.93 (0.84, 1.03)	
Senior high shool or above	148	765	0.90 (0.86, 0.94)	
**Maternal occupation**				0.563
In employment	122	747	0.93 (0.89, 0.97)	
Without employment	96	201	0.91 (0.85, 0.97)	
**Residence**				<0.001
Urban	136	679	0.88 (0.83, 0.93)	
Rural	82	269	0.96 (0.91, 1.02)	
**Parity**				0.167
0	123	761	0.92 (0.88, 0.96)	
≥ 1	95	187	0.92 (0.87, 0.98)	
**Medication use**				0.346
No	126	660	0.93 (0.89, 0.97)	
Yes	92	288	0.90 (0.85, 0.95)	
**Anemia**				0.752
No	181	845	0.91 (0.88, 0.95)	
Yes	37	103	0.94 (0.86, 1.03)	
**Overall**	218	948	0.92 (0.89, 0.95)	

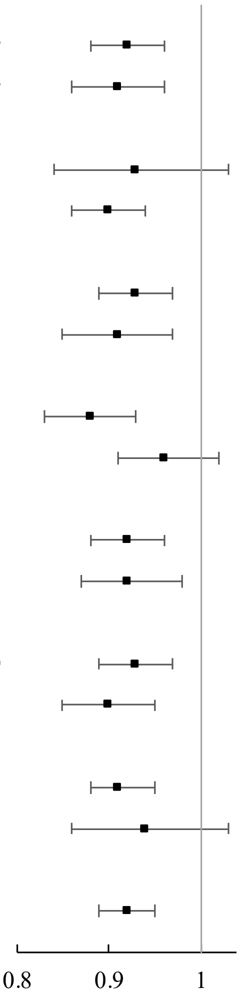

Figure S3. Subgroup analyses for the relationship between per 1 higher in maternal oxidative balance score in pregnancy and atrial heart defects. Analyses were adjusted for total energy intake, maternal age, education, occupation, residence, parity, medication use, and anemia.

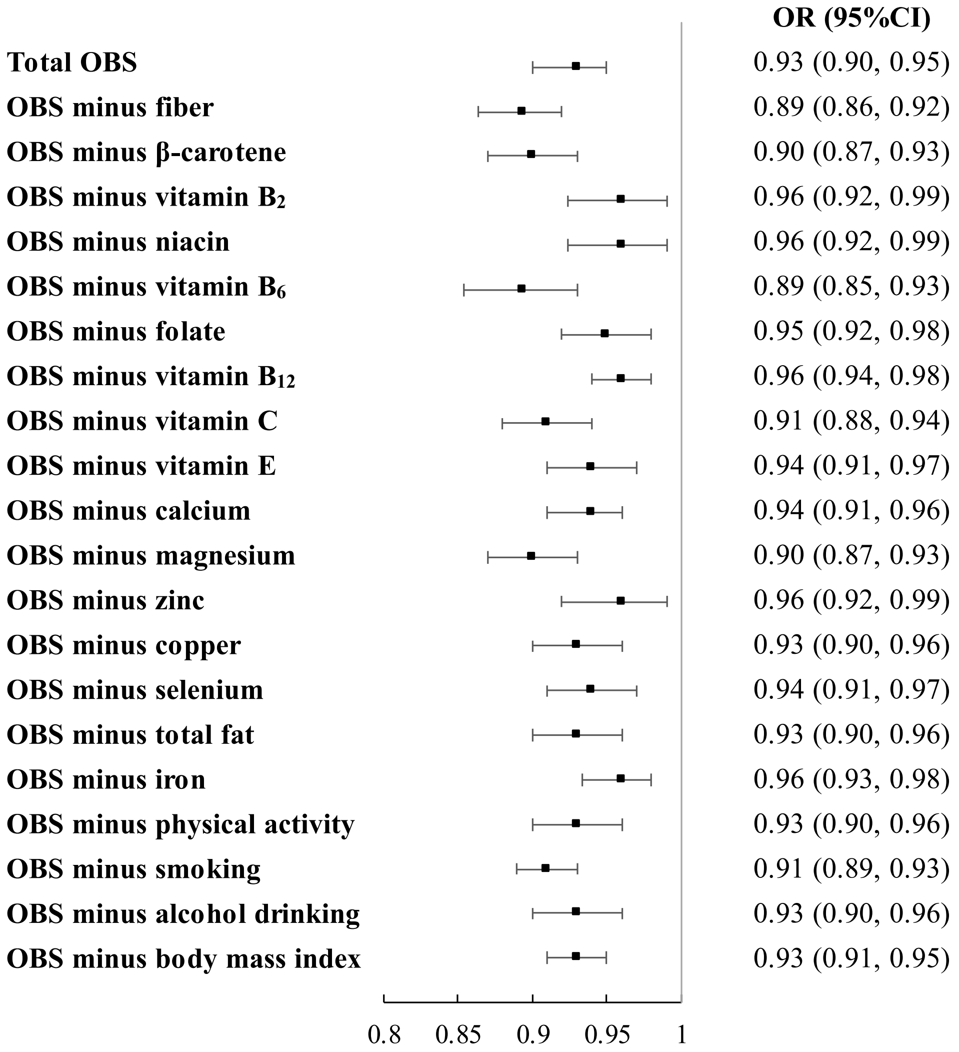

Figure S4. The relationship between per 1 higher in maternal oxidative balance score in pregnancy and total congenital heart defects after alternate subtraction of each component. OBS, oxidative balance score. Analyses were adjusted for total energy intake, maternal age, education, occupation, residence, parity, medication use, anemia, and corresponding subtracted component.

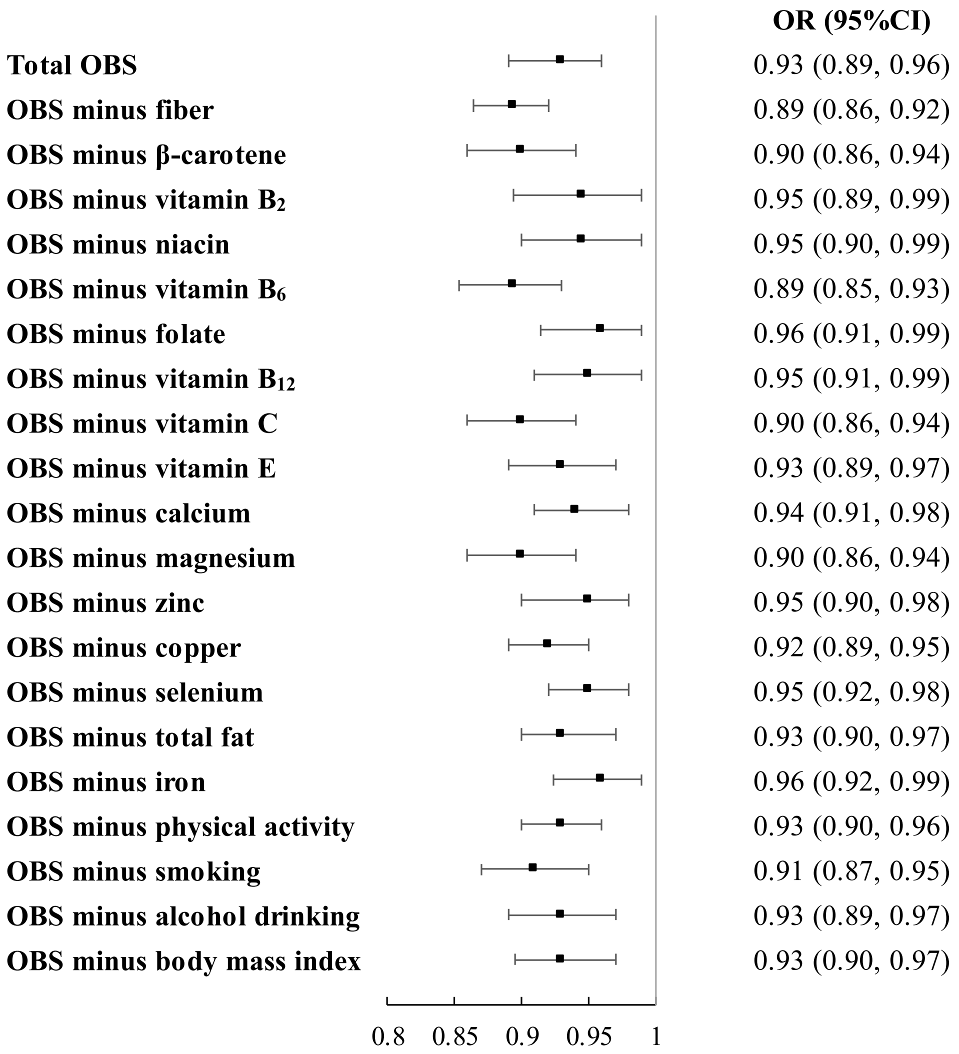

Figure S5. The relationship between per 1 higher in maternal oxidative balance score in pregnancy and ventricular heart defects after alternate subtraction of each component. OBS, oxidative balance score. Analyses were adjusted for total energy intake, maternal age, education, occupation, residence, parity, medication use, anemia, and corresponding subtracted component.

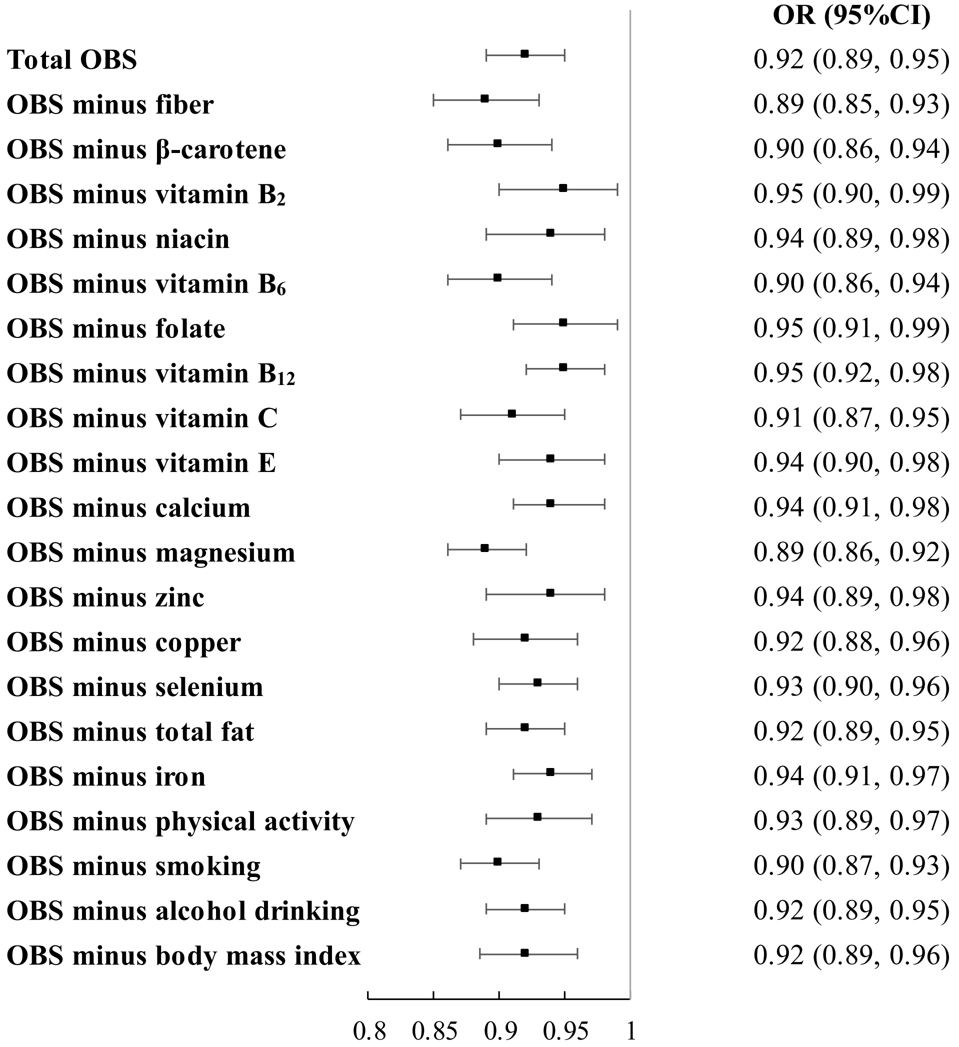

Figure S6. The relationship between per 1 higher in maternal oxidative balance score in pregnancy and atrial heart defects after alternate subtraction of each component. OBS, oxidative balance score. Analyses were adjusted for total energy intake, maternal age, education, occupation, residence, parity, medication use, anemia, and corresponding subtracted component.
